# FIT2DRIVE: DEVELOPMENT AND TESTING OF A PREDICTOR OF DRIVING CAPACITY OF OLDER ADULTS WITH COGNITIVE CONCERNS

**DOI:** 10.1093/geroni/igac059.1243

**Published:** 2022-12-20

**Authors:** Ruth Tappen, David Newman, Monica Rosselli, Joshua Coniff

**Affiliations:** Florida Atlantic University, Boca Raton, Florida, United States; Florida Atlantic University, Boca Raton, Florida, United States; Florida Atlantic University, Davie, Florida, United States; Florida Atlantic University, Davie, Florida, United States

## Abstract

The decision to stop driving has been reported by caregivers of persons with dementia as one of the most difficult ones they confront. Additionally, primary care providers to whom they often turn for guidance report being unprepared to provide them with evidence-based information. Our aim was to develop and test a predictive model employing 2 or 3 brief, easily administered cognitive tests to predict the individual’s likelihood of passing an on road driving test. Participants were licensed drivers recruited from our Memory Center’s driver evaluation program and the community to obtain a broadly representative sample of older drivers. A total of 357 drivers age 60 to 97 (mean 81) completed an established on-road driving test and battery of short cognitive tests. Two-thirds of the sample were white, non-Hispanic, one third were Black, African American, 59% male and 41% female, mean MMSE score of 24. Employing Receiver Operating curve analysis, the best set of predictors included participant age, MMSE utilizing world spelled backwards (a better predictor than serial 7’s), Trails B time in seconds and participant age yielding 95% AUC (area under the curve). The model was invariant across gender, education and ethnic group. A website with an interactive calculator in which this data is entered and likelihood of passing an on-road driving test prediction is presently under construction and will be available first to providers and later to individuals with a concern about continuing capacity to drive. Funded by Florida Department of Health Ed and Ethel Moore Alzheimer’s Disease Initiative.

